# Synthesis of IL-6 by Hepatocytes Is a Normal Response to Common Hepatic Stimuli

**DOI:** 10.1371/journal.pone.0096053

**Published:** 2014-04-24

**Authors:** Callie A. Norris, Mu He, Liang-I Kang, Michael Qi Ding, Josiah E. Radder, Meagan M. Haynes, Yu Yang, Shirish Paranjpe, William C. Bowen, Anne Orr, George K. Michalopoulos, Donna B. Stolz, Wendy M. Mars

**Affiliations:** 1 Department of Pathology, University of Pittsburgh, School of Medicine, Pittsburgh, Pennsylvania, United States of America; 2 Department of Cell Biology and Physiology, University of Pittsburgh, School of Medicine, Pittsburgh, Pennsylvania, United States of America; Agency for Science, Technology and Research - Singapore Immunology Network, Singapore

## Abstract

Exogenous interleukin 6 (IL-6), synthesized at the initiation of the acute phase response, is considered responsible for signaling hepatocytes to produce acute phase proteins. It is widely posited that IL-6 is either delivered to the liver in an endocrine fashion from immune cells at the site of injury, or alternatively, in a paracrine manner by hepatic immune cells within the liver. A recent publication showed there was a muted IL-6 response in lipopolysaccharide (LPS)-injured mice when nuclear NFκB was specifically inactivated in the hepatocytes. This indicates hepatocellular signaling is also involved in regulating the acute phase production of IL-6. Herein, we present extensive *in vitro* and *in vivo* evidence that normal hepatocytes are directly induced to synthesize IL-6 mRNAs and protein by challenge with LPS, a bacterial hepatotoxin, and by HGF, an important regulator of hepatic homeostasis. As the IL-6 receptor is found on the hepatocyte, these results reveal that induction of the acute phase response can be regulated in an autocrine as well as endocrine/paracrine fashion. Further, herein we provide data indicating that following partial hepatectomy (PHx), HGF differentially regulates IL-6 production in hepatocytes (induces) versus immune cells (suppresses), signifying disparate regulation of the cell sources involved in IL-6 production is a biologically relevant mechanism that has previously been overlooked. These findings have wide ranging ramifications regarding how we currently interpret a variety of *in vivo* and *in vitro* biological models involving elements of IL-6 signaling and the hepatic acute phase response.

## Introduction

IL-6 is a key mediator of the acute phase response. Additionally, IL-6 plays a central role in restoring normal hepatic function following liver injury [1], [2]. During a general acute phase response, IL-6, produced by immune cells at the site of injury, is deemed to be one of the primary factors that signals liver hepatocytes to produce acute phase proteins [3]. The predominant belief is that IL-6 is released into the circulation, taken up by the liver, and the resident hepatocytes then recognize the IL-6 as a stimulus to begin production of acute phase proteins. Similarly, a principal hypothesis has developed positing that in situations where an injury is inflicted directly upon the liver it is the resident immune cells, such as the Kupffer cells (hepatic macrophages), that primarily produce the IL-6 used for stimulating acute phase protein production. These hepatic immune cells are considered to be the exclusive providers of the IL-6 that subsequently signals hepatocytes in the damaged liver to produce acute phase proteins while helping to restore hepatic function [1], [2]. Still, although both IL-6 and these internal immune cells are important for hepatic regeneration [4], [5], an exact understanding of how the two contribute to hepatic repair is still wanting.

LPS injection is often used to mimic systemic, gram-negative bacterial infections that induce an acute phase response, and IL-6 mRNA production is known to subsequently occur under the transcriptional control of NFκB [6], [7]. Maeda *et al*. established that when NFκB function is selectively inactivated in hepatocytes, IL-6 mRNA production is severely muted in whole livers obtained from mice at 4 h after injection of LPS [6]. As the time frame in which the observed the changes in IL-6 was rapid, and the induced NFκB defect was hepatocyte-specific, these experiments suggested to us that hepatocytes, in addition to immune cells, might also serve as a direct source of IL-6 during an acute phase reaction, i.e. the hepatic response might also have an autocrine component. Previously, it was demonstrated that transplantation of wild type bone marrow into IL-6 deficient animals is able to rescue normal liver function in response to targeted hepatic injury, proving the importance of immune cells in providing IL-6 to hepatocytes [8]. However, importantly, these marrow transplantation experiments did not rule out the possibility that other, non-immune cell types might also be capable of producing IL-6. Furthermore, although production of IL-6 by hepatocytes has not specifically been demonstrated, hepatocytes have been shown to produce other cytokines in response to LPS treatment [9].

We now show that primary hepatocytes cultured under completely serum-free conditions can synthesize both IL-6 mRNAs and protein, and that the levels of IL-6 in these cultures are subject to regulation by both LPS and HGF. *In vivo*, our studies show that hepatocytes are capable of synthesizing both IL-6 mRNAs and protein following injury induced by LPS injection as well as after hepatic resection. In the latter model, specific loss of the HGF receptor (MET) in hepatocytes results in the anticipated decrease in hepatic IL-6; however, global hepatic loss (all cell types) leads to a relative increase with a concomitant enhanced staining of IL-6 in a limited immune cell population. As we have previously shown HGF suppresses LPS-induced production of IL-6 in cultured macrophages via the MET receptor [10], it appears likely that following a 70% PHx, HGF can suppress the macrophage-mediated production of IL-6 while simultaneously inducing it in hepatocytes. Importantly, this indicates that under the appropriate circumstances, regulation of the endogenous hepatocellular IL-6 receptor can be autocrine instead of endrocrine/paracrine [11].

## Results

### Primary rat hepatocytes produce IL-6 mRNA and protein

**Figure 1:**
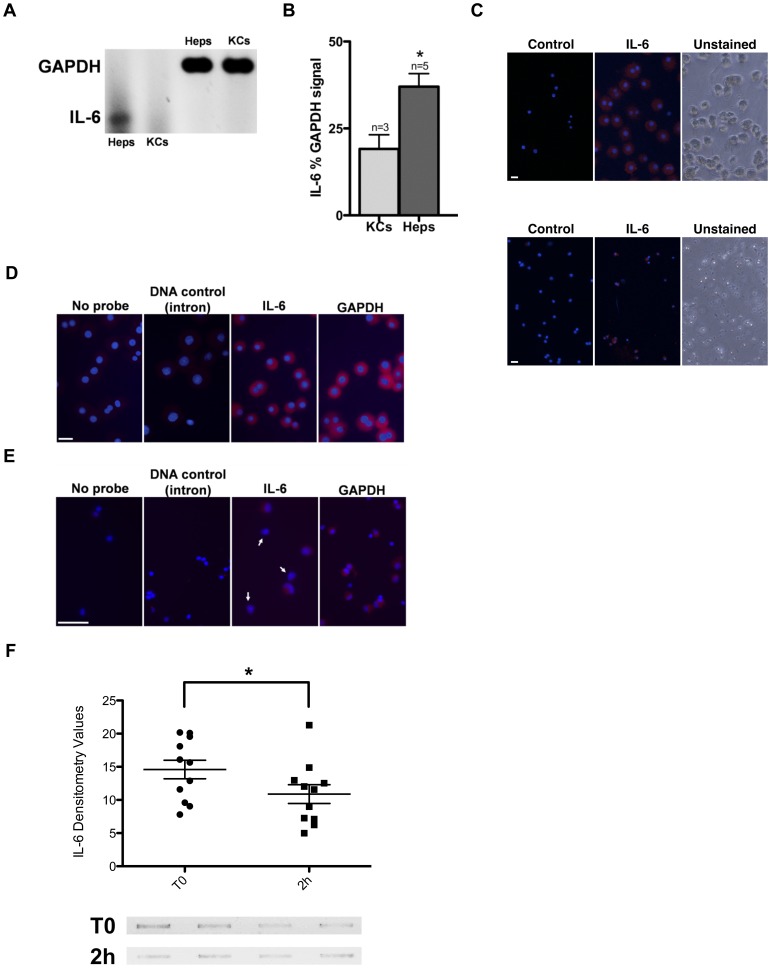
IL-6 in serum-free cultured hepatocytes and Kupffer Cells. (A) Representative RT-PCR depicting IL-6 from a serum-free rat hepatocyte culture (Heps) at 2 h after attachment (also see Figure 4A) and from fresh Kupffer Cells (KCs) at 15 min after attachment. GAPDH was used as a positive control. Cells are from the same animal. (**B**) Densitometric analyses depicting percent IL-6 mRNA, as compared to GAPDH, with mean ± s.e.m., in hepatocytes and Kupffer cells. *n*  =  number of independent trials using separate animals. * indicates statistical significance, *P* = 0.0238, between hepatocytes and Kupffer cells, two-tailed t-test. (**C**) Immunofluorescent staining showing IL-6 in rat hepatocytes (top, note the comparatively large size and presence of typical bi-nucleated cells) and Kupffer cells (bottom) plated completely serum-free from the same animal. Secondary antibody was conjugated with Cy3 (red). The left panel is a control visualized at the same gating with no primary antibody added. The right panel is a phase contrast image of the cells taken at the same magnification just prior to staining. (**D, E**) mRNA FISH depicting IL-6 in hepatocytes (**D**), or Kupffer cells (**E**) from the same animal. Arrows indicate IL-6 mRNA-negative Kupffer cells. Animal *n* = 8. Nuclei were stained with Hoescht dye (blue). Probes were conjugated with Alexa 546 (red). Left to right, hybridization with: reagent control (no probe, used for gating), negative control (labeled IL-6 intron), labeled IL-6 cDNA and labeled GAPDH cDNA (positive control). (**F**) Representative slot blot samples (bottom) and analyses (top, n = 12) of media from freshly plated hepatocytes at 2 h after attachment (T0) or after another 2 h (2 h). * *P* = 0.0313 significance, using paired two-tailed t-test. Scale bars, 20 µm in images.

To initially test our hypothesis that hepatocytes can make IL-6, purified hepatocytes from rat livers were isolated and plated in primary cultures using completely serum-free conditions. Cell extracts were then assayed for IL-6 mRNAs (RT-PCR) and protein (western blot). Figure 1 indicates that both are present, with hepatocytes showing low but consistent levels of IL-6 mRNAs (Figures 1A, B) and mature protein (Figure 1C). To ascertain whether the IL-6 was coming from contaminating macrophages in the hepatocyte cultures, Kupffer cells were simultaneously isolated and tested for mRNA and protein production. Surprisingly, IL-6 message in purified Kupffer cell extracts was only about 50% that of hepatocyte cultures (Figures 1A, B). To verify these results and identify the individual cells producing the protein and mRNA, we next conducted immunofluorescence and mRNA fluorescent *in situ* hybridization (FISH) studies on freshly plated cells from serum-free hepatocyte cultures. Figure 1C illustrates that IL-6 protein is present in the majority of newly cultured hepatocytes although not in all of the freshly plated Kupffer cells. mRNA FISH verifies the protein is likely coming from internal production of endogenous IL-6 message (Figure 1D). In concert with the protein results, isolated Kupffer cells, cultured and subjected to mRNA FISH at the same time as the hepatocytes were heterogeneous, displaying fewer positive cells overall than hepatocytes (Figure 1E) and validating the results obtained from the RT-PCR assays. Finally, we tested to see if the IL-6 protein is secreted from the serum free hepatocyte cultures (Figure 1F). IL-6 was present in media from the freshly plated cells and significantly decreased over time.

### Hepatocytes express IL-6 mRNA after PHx

**Figure 2:**
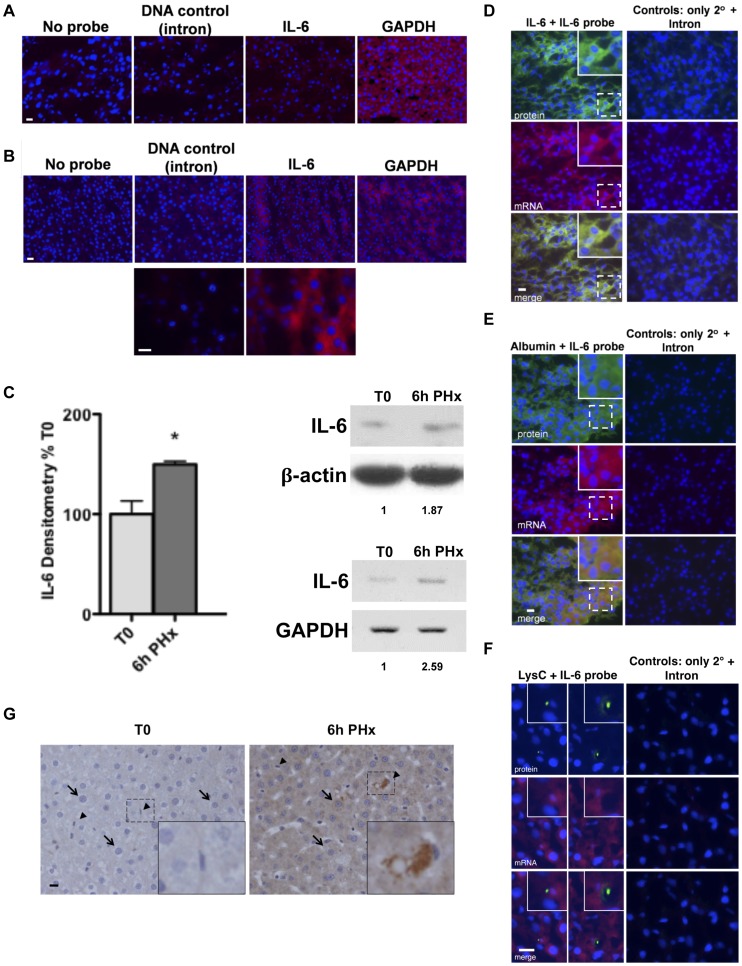
IL-6 production by hepatocytes after PHx. Resting rat livers (A) or remnant livers removed 6 h after PHx (**B**) were probed for IL-6 mRNA by FISH. Probes, red; Nuclei, blue. Lower panel in B represents a higher magnification from the panel above it. Panels in A and B were taken from hybridizations performed the same day and those at the lower magnifications were gated the same for the picture. Mock hybridizations (no probe) were used as the immunofluorescent gating control. (**C**) Densitometric analysis of IL-6 on western blots using protein lysates prepared from resting livers (T0) or remnant livers at 6 h post-PHx. Actin was used as a reference control. Animal *n* = 3. * indicates statistical significance (*P* = 0.0217), two way t-test. On the right is a representative western blot and RT-PCR from a single animal's whole liver showing the relative changes in protein and mRNA when compared to control (actin for protein, GAPDH for mRNA). Numbers underneath are the numerical change relative to resting liver (T0). (**D, E, F**) Simultaneous staining for IL-6 (**D**), albumin (**E**), or lysozyme C (LysC) (**F**) proteins (green) and IL-6 mRNA (red) in livers at 6 h after PHx. Co-localization (merge) appears as yellow. Left panels were probed with the IL-6 cDNA and antibody against IL-6, albumin, or lysozyme C. Right panels were probed with the IL-6 intron and secondary antibody only (controls). For **D** and **E**, broken line boxes represent the areas magnified in the insets. For **F**, the initial magnification shown is higher and 2 separate regions containing macrophages have been magnified. Controls were used for gating levels. (**G**) Immunohistochemical staining for IL-6 in resting rat livers or remnant livers removed 6 h after PHx. Arrows and arrowheads point to hepatocytes and macrophages, respectively. Inset shows magnification of one of the macrophage arrowheads. Scale bars, 20 µm in images.

We next tested to see whether IL-6 is also produced by hepatocytes *in vivo* in response to direct hepatic injury. The PHx model of rat liver regeneration was utilized for these studies due to the well-documented timing of surgically induced changes. In remnant livers removed after 70% PHx, enhanced phosphorylation of MET, the HGF receptor, is detected from 5 to 60 min after resection [12], NFκB translocation is evident as early as 30 min after resection [13], and IL-6 mRNA levels begin to increase by 2 h, remaining elevated for 24 h [14]. We anticipated that in this particular model, if IL-6 expression is hepatocellular, the mRNAs should be apparent in the hepatocytes of remnant livers during the time frame between 2 and 24 h after surgical resection. As shown in Figure 2A, in resting livers there were minor quantities of detectable IL-6 mRNAs in the organ under the conditions of our assay. However, in livers we tested at 6 h after PHx, when the circulating levels of IL-6 protein become elevated [14], IL-6 mRNAs were readily apparent across the tissue (Figure 2B), correlating with a general increase in hepatic IL-6 protein and RNA (Figure 2C). Double staining for IL-6 mRNAs and IL-6 protein demonstrated the individual cells producing the message also harbor the protein (Figure 2D). The majority of these cells are hepatocytes, as verified by double staining using the marker albumin (Figure 2E); however, positive staining in macrophages is also observed (Figure 2F). An increase in IL-6 staining, relative to resting liver, could also be observed in both hepatocytes and macrophages by immunohistochemistry (Figure 2G).

### Hepatocytes express IL-6 mRNA in response to LPS *in vitro* and *in vivo*

**Figure 3:**
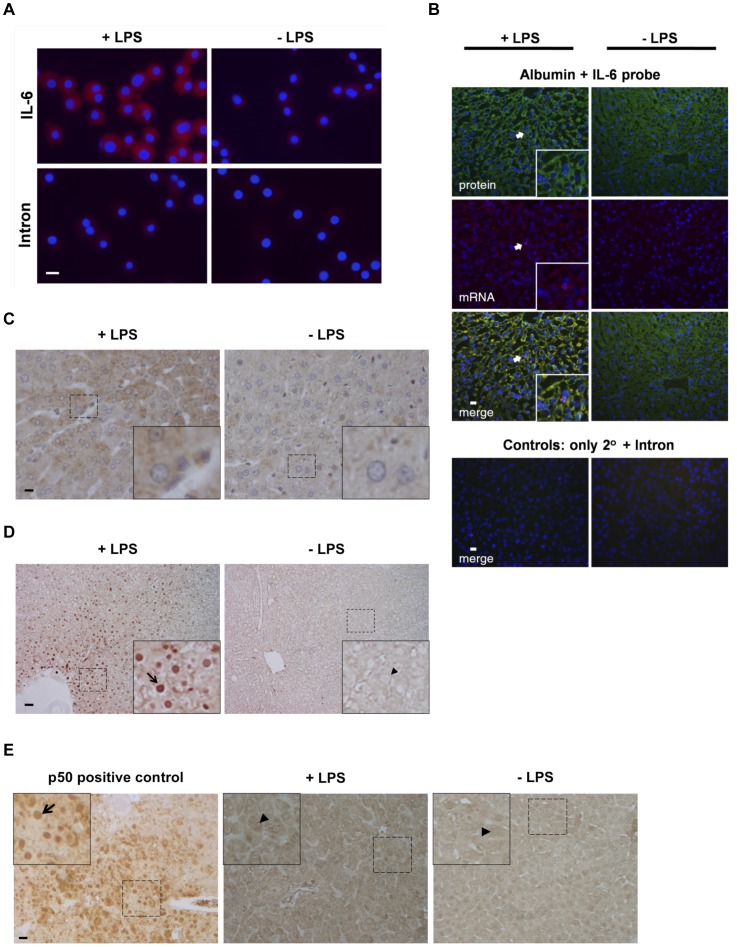
IL-6 synthesis and NFκB signaling in hepatocytes after LPS injection. (A) FISH for IL-6 mRNAs in serum-free rat hepatocyte cultures, 15 min after media change with 1 µg LPS/ml or diluent (control). For these photographs, as a baseline level of IL-6 mRNA was known to be present (see 1D), gating was adjusted with the diluent-treated sample serving as the baseline. (**B, C**) Rat livers were injected with 100 µg/kg LPS or saline (control) and harvested at 4 h post treatment. In **B**, samples were simultaneously stained for albumin protein (green) and IL-6 mRNA (red). Co-localization (merge) appears as yellow. A presumptive inflammatory cell (expressing IL-6 mRNA but albumin-negative) is indicated by an arrow and featured in magnified inserts. Mock hybridizations (not shown) were used as the immunofluorescent gating control. **C** shows standard immunohistochemical staining using an antibody against IL-6. (**D, E**) Representative immunohistochemistry depicting nuclear p65 (**D**), or p50 (**E**) with and without LPS treatment. Arrows show nuclei stained with p65 or p50 (brown), arrowheads indicate unstained nuclei (colorless). Simultaneously stained tissue section from liver of ILK-null mice [40] shows positive nuclear localization staining for p50 (left panel of **D**). Dotted boxes are featured in magnified inserts. Scale bars, 20 µm in all images except for **D** (40 µm).

Administration of LPS can be used to mimic induction of a systemic acute phase response. As hepatocytes have toll-like receptors that allow NFκB signaling to be invoked after stimulation with LPS [9], and as NFκB can act upstream of IL-6 to control synthesis [6], [7], we next tested to see whether LPS can directly up-regulate IL-6 production in primary rat hepatocytes. As shown in Figure 3A, IL-6 mRNAs were substantially increased very early in response to LPS using our serum-free culture system. To determine whether up-regulation of IL-6 by hepatocytes can also occur *in vivo*, we next tested for the presence of hepatocellular IL-6 mRNAs following LPS injection using the time frame of Maeda *et al* [6]. As shown in Figure 3B (mRNA and merge panels), at 4 h following LPS administration, IL-6 mRNAs are readily detectable, whereas resting hepatocytes from animals injected with a saline control harbor minimal quantities of IL-6 message. Double staining with an antibody against albumin definitively identifies a majority of the cells expressing IL-6 as hepatocytes, although occasional positive immune cells were also observed (Figure 3B). This increase was verified using immunohistochemistry as well (Figure 3C). We also verified there was NFκB signaling at 1 h after LPS injection, when translocation of NFκB peaks after LPS administration in mice [15]. An increase in nuclear staining for the NFκB p65 subunit was found in hepatocytes from the LPS-injected animals, relative to saline-injected controls, with staining especially strong in patches throughout the tissue and around hepatic vessels (Figure 3D). Our p65 data supports the finding that there was a paucity of nuclear NFκB reported by Maeda *et al* when nuclei from hepatocyte-specific NFκB knockouts, versus their control counterparts, were subjected to EMSA at 1 h following LPS injection [6]; however, surprisingly, we were unable to detect nuclear staining for p50, the classical NFκB partner of p65, in either treated or untreated animals (Figure 3E).

### HGF regulates IL-6 production in hepatocytes

**Figure 4:**
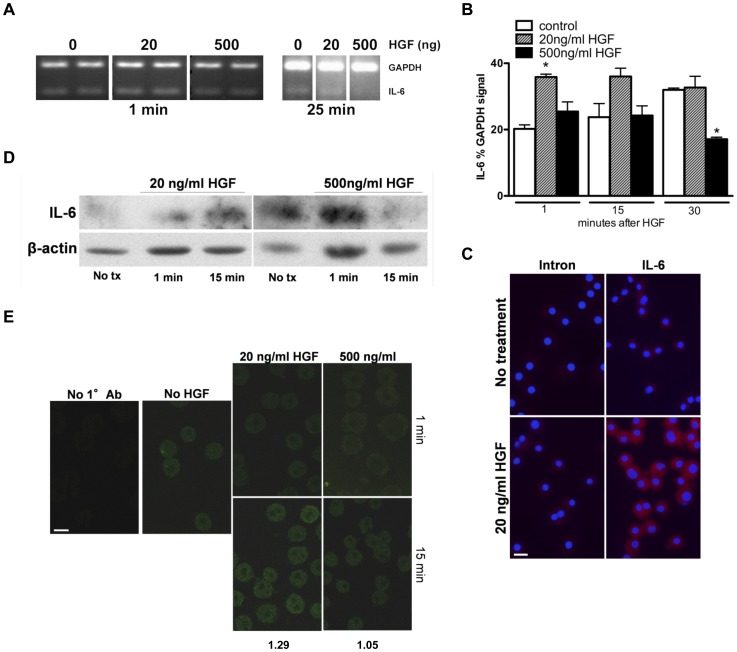
HGF-mediated effects on IL-6 production in serum-free hepatocyte cultures. (A) Representative RT-PCR of IL-6 and GAPDH (control) in serum-free hepatocyte cultures over 25 min, in the presence or absence of 20 or 500 ng HGF/ml. Duplicates shown are separate cultures from a single animal. (**B**) Summary graph of relative percent IL-6 mRNA to GAPDH in hepatocytes treated with 20 or 500 ng HGF/ml at 1, 15 and 30 min, with mean ± s.e.m. *n* = 4. * indicates statistical significance, *P*<0.05, between time point control and designated condition. (**C**) FISH probing for IL-6 mRNAs in serum-free hepatocyte cultures at 15 min after media change with either 20 ng HGF/ml (bottom panel) or diluent (no treatment, top panel). Mock hybridizations (not shown) were used as the immunofluorescent gating control. (**D**) Representative western blots for detection of IL-6 in serum-free hepatocyte cultures over a 15 min time period, in the absence or presence of 20 or 500 ng HGF/ml. Animal *n* = 3. (**E**) Immunofluorescent staining for IL-6 in serum-free hepatocyte cultures over 15 min, in the presence or absence of 20 or 500 ng HGF/ml. The cells without primary antibody were used as the immunofluorescent gating control. Numbers under figure represent quantifiable increase at 15 min, relative to 1 min staining shown directly above. Scale bars, 20 µm in all images.

**Figure 5:**
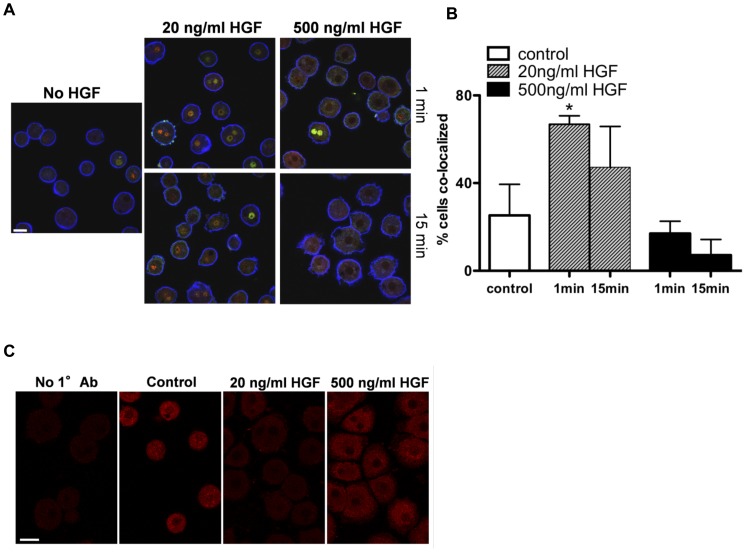
NFκB signaling corresponds to changes in IL-6 production in response to HGF. (A) Confocal staining for the NFκB subunits p50 and p65 in serum-free hepatocyte cultures over 15 min, in the presence or absence of 20 or 500 ng HGF/ml. Actin staining (phalloidin) of the plasma membrane is shown in blue; p50, red; p65, green; with co-localization appearing as yellow. The control cells (no HGF) were used as the immunofluorescent gating control. (**B**) Summary graph of the percent nuclear staining for NFκB (co-localized staining for p50 and p65) in the experiment shown in A, with mean ± s.e.m. *P* = 0.0284, significant for one way ANOVA. * indicates statistical significance, *P*<0.05 from control, Newman-Keuls test. (**C**) Immunofluorescent staining for the NFκB inhibitor, IκB, in serum-free hepatocyte cultures from the experiment shown in A, at 15 min after stimulation with 0 (control), 20 or 500 ng HGF/ml. The cells without primary antibody were used as the immunofluorescent gating control. Scale bars, 20 µm in all images.

We were next interested in knowing if factors other than LPS could stimulate production of IL-6 in hepatocytes in an NFκB -dependent manner. HGF, a primary mitogen for hepatocytes, has also been reported to mediate its effects in part via the NFκB pathway [16]. Hence, we next determined whether HGF can also affect IL-6 production in hepatocyte cultures, and whether this correlates with concomitant changes in the NFκB pathway. Assays for IL-6 mRNA levels *in vitro* focused on the first 30 min after HGF stimulation due to the extremely short half-life of IL-6 mRNAs that is directly related to message stability [17]. GAPDH, with a half-life of at least 8 h [18], served as an internal control. The addition of mitogenic doses of HGF (20 ng/ml) resulted in a transient and significant enhanced level of IL-6 mRNAs (Figures 4A–C). Since high doses of HGF can suppress mitosis [19] or induce apoptosis [20], for control purposes we also tested a non-mitogenic dose of HGF (500 ng/ml, efficacy confirmed by lack of thymidine incorporation, data not shown). In contrast to the mitogenic dose of HGF, when the higher amount of HGF was administered there was a significant decrease of IL-6 mRNAs over time, likely reflecting its short half-life (Figures 4A–B). As anticipated, results with the IL-6 mRNAs were also followed by rapid fluctuations in the quantity of IL-6 protein as shown by both western blot and immunofluoresence (Figures 4D, E). Concurrently, we examined whether the HGF-induced alterations in IL-6 levels corresponded with the expected outcomes for localization of NFκB and its regulatory inhibitor, IκB. Individual staining for the NFκB subunits, p50 and p65, showed that at mitogenic doses, HGF elicited a significantly enhanced nuclear localization of the NFκB dimer (Figures 5A, B). Enhanced translocation of NFκB to the nucleus corresponded with an associated loss of cytoplasmic IκB (Figure 5C), the protein that retains the NFκB dimer in its inactive, cytoplasmic configuration. Conversely, when hepatocytes were exposed to the non-mitogenic dose of HGF, no significant changes were noted in the locations of p50, p65, or IκB (Figures 5A, C), in accordance with the inability of this HGF dose to increase IL-6 production.

### Hepatocyte-specific loss of HGF signaling abrogates IL-6 production after PHx

**Figure 6:**
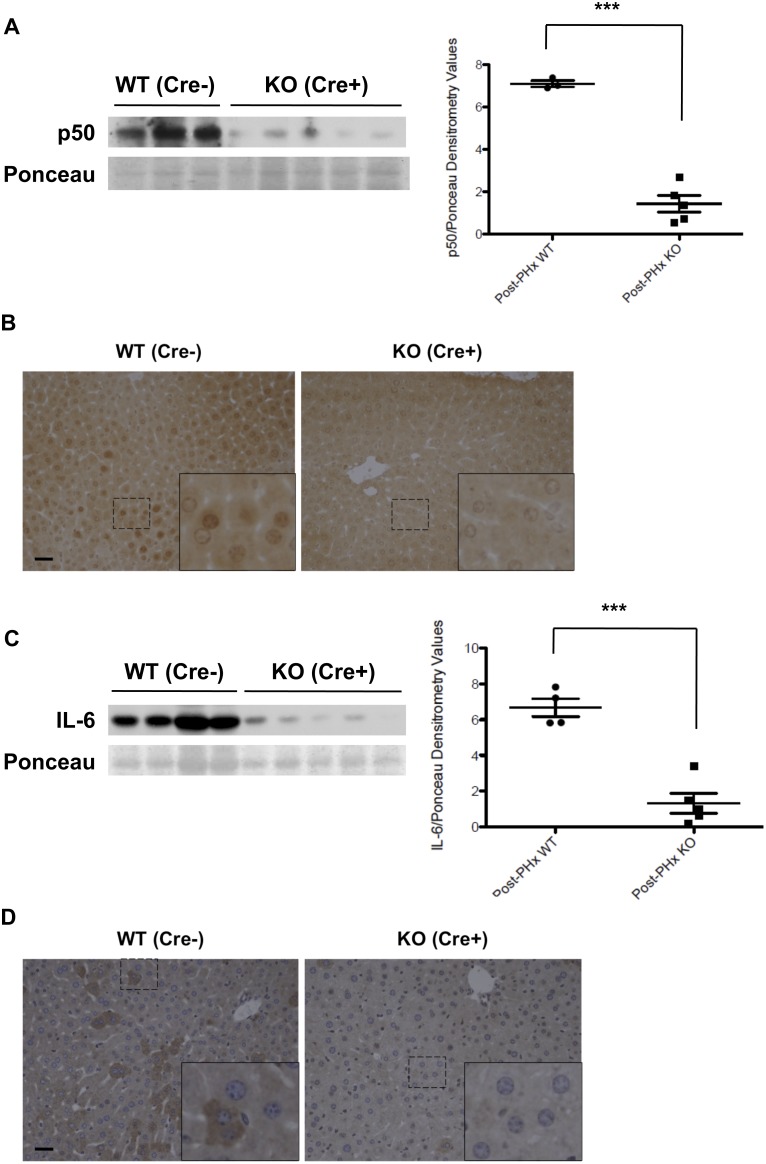
Hepatocyte-specific HGF-mediated effects on IL-6 production in mice after PHx. (A) Western blot analysis of hepatic NFκB p50 protein expression at 6 h post-PHx in MET flox mice either lacking Cre recombinase (WT) or possessing Cre (KO) is presented, using nuclear-enriched lysates. A representative Ponceau Red-stained protein band, stained prior to antibody probing, is shown for loading comparison. Ponceau-normalized densitometry comparing WT to KO levels of p50 is shown at right. *P*<0.0001, significant, for two-tail Student's t-test. *** indicates statistical significance, *n* = 3–5. (**B**) Standard immunohistochemical staining using the same antibody against p50 as used for the western blot shown in **A**. Livers removed at 6 h post-hepatectomy from WT (Cre−) or KO (Cre+) animals are shown. Counterstain was deliberately omitted to enhance visualization. Insets represent magnifications of the figure in order to better see the hepatocellular nuclei. (**C**) Western blot analyses of hepatic IL-6 protein expression in cytoplasmic-enriched lysates for the 6 h PHx experiment described in subfigure A. Densitometric analysis of IL-6 protein expression was performed as described in subfigure A. *P* = 0.0002, significant, for two-tail Student's t-test. *** indicates statistical significance, *n* = 4–5. (**D**) Standard immunohistochemical staining using an antibody against IL-6. Livers extracted at 6 h post-hepatectomy from WT (Cre−) or KO (Cre+) animals are depicted. Insets represent magnifications of the figure in order to better see the hepatocytes and a presumptive macrophage. Scale bars, 20 µm in all images.

HGF signaling through its receptor, MET, is an early *in vivo* response following PHx [12]. To determine if the rise in hepatocellular IL-6 we detected after PHx (see Fig. 2) occurs *in vivo* in response to MET activation, we next performed resections on mice where Cre-Lox technology was used to specifically remove MET from the hepatocytes. Removal of MET was confirmed by western blot (data not shown) with subsequent abrogation of NFκB-p50 nuclear translocation in the remnant liver at 6 h post-resection (Figures 6A and 6B). In the hepatocellular targeted mice we observed the expected abrogation of IL-6 protein at 6 h post-surgery (Figures 6C and 6D), indicating that HGF signaling through hepatocellular MET is necessary for the increase in hepatic IL-6 that is observed after PHx.

### Loss of HGF signaling enhances IL-6 production in immune cells

**Figure 7:**
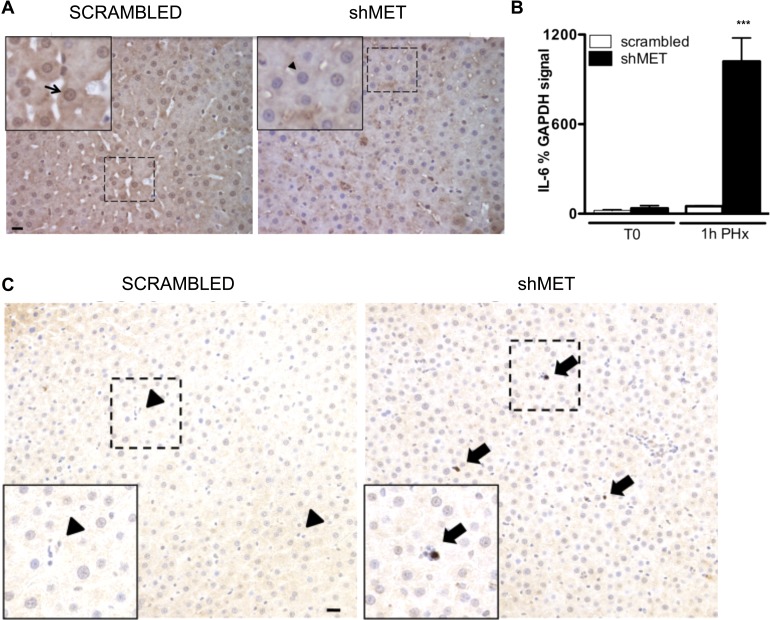
Effects of non-specific loss of MET on IL-6 production after PHx in rats. Livers from rats pre-treated for 24 h with shRNAs to inactivate the HGF receptor (shMET) or control shRNAs (scrambled) were removed at time 0 (T0) or at 1 h after PHx and analyzed for p50 and IL-6. (**A**) Representative immunohistochemistry depicting nuclear p50 in the shRNA treated animals (scrambled and shMET) at 1 h after PHx. Arrows show nuclei stained with p50 (brown), arrowheads indicate unstained nuclei (counterstain, blue). Scale bars, 20 µm. (**B**) Densitometric analyses of percent IL-6 mRNA, compared to percent GAPDH control, with mean ± s.e.m., in livers from shRNA-treated animals. *P* = 0.0001, significant for one way ANOVA. *** indicates statistical significance, *P*<0.05, from all other samples, Newman-Keuls test. (**C**) Representative images of immunohistochemistry for IL-6 in the livers of shRNA treated animals at 1 h after PHx. Arrows show IL-6 positive immune cells (stained brown), arrowheads indicate unstained immune cells. Scale bars, 20 µm in all images.

Finally, we wondered if the cell source of IL-6 might be relevant with regard to overall hepatic health. Mice with hepatocellular-targeted deletion of MET are reported to have a high death rate at 48 h following PHx whereas surprisingly, mice with a whole body deletion of the receptor have been reported to survive [21], [22]. This suggests that in hepatocyte-specific knockouts, MET-mediated signaling in the non-parenchymal cells may somehow be deleterious to hepatic health. In a recent publication we demonstrated that unlike what we now see with hepatocytes, HGF instead suppresses IL-6 production in macrophages via signaling through the MET receptor [10]. Further, unlike what we observed in our WT animals (especially rats, see Figure 2G), we did not readily observe morphologically identifiable activated macrophages staining for IL-6 in the livers of animals where MET was specifically removed from the hepatocytes. As IL-6 is an important promoter of hepatocyte viability after PHx [23], we hypothesized that the reason animals with global loss of MET fair better than mice with hepatocyte targeting is because the additional MET deficiency in immune cells subverts the HGF-mediated suppression of IL-6, allowing for an enhanced production of IL-6 that can then rescue the animals. In concordance with this hypothesis, Borowiak *et al*. reported that whole body MET knockout animals do display a marked elevation of IL-6 following PHx when compared to their wild type controls [22]. To test if removing MET from just the liver, as opposed to the whole body, would invoke a similar effect, rats were pre-treated with shRNAs through the superior mesenteric vein to specifically decrease MET in whole livers, prior to performing hepatic resections. Previously we reported that under these conditions, MET is transiently suppressed and regeneration is delayed in the animals. Specifically, at 24 h after shRNA injection (when the surgeries are performed) MET mRNA levels are reduced ∼1.5 fold, and tyrosine-phosphorylated MET protein levels are decreased ∼3 fold [24]. As anticipated, examination of the p50 subunit indicated that translocation of NFκB to the nucleus was impaired at 1 h after PHx in the shRNA-treated animals (Figure 7A). However, despite the decrease in NFκB translocation, there was an early and significant increase, rather than a decrease, in the quantity of IL-6 mRNA at 1 h post-PHx, when overall hepatic MET was suppressed (Figure 7B). To determine if this increase in IL-6 was due to immune cells producing more IL-6, tissues were stained for IL-6. As shown in Figure 7C, strong staining is observed in a sub-population of hepatic immune cells, albeit ones that do not appear to be the classically activated macrophages, at 1 h post-PHx in the shRNA-treated, but not scramble-treated, animals. This indicates there is a sub-population of immune cells can be a source of increased IL-6 production when overall MET is suppressed.

## Discussion

There is a long history to the prevailing theory that immune cell-generated IL-6 induces hepatocytes to manufacture acute phase proteins [3], [4], [8]. Generally, it is thought that when an injury occurs, no matter where the insult initiates, an immune reaction is generated in response to the damage. This ultimately results in IL-6 production and secretion, with the IL-6 then travelling to the liver via the circulation. In cases where the injury occurs to the liver itself, instead of releasing the IL-6 into the general circulation, the endogenous immune cells present in the liver are thought to act in a paracrine manner and provide IL-6 to their neighbouring hepatocytes. Although the stimulus generating this immune cell-mediated production of IL-6 within the liver is not always apparent, for the PHx model of hepatic repair, there is some evidence suggesting that products from natural bacterial flora within the gut are released during the surgical manipulations. It is thought that these stimulants then induce Kupffer cells to produce IL-6 [25] and that subsequently, hepatocytes respond in a paracrine manner to the IL-6 via their endogenous IL-6 receptor [11]. Importantly, while our findings do not refute the hypothesis that hepatocellular signaling can occur in response to exogenously produced immune cell IL-6, we are now able to definitively demonstrate that hepatocytes themselves can also synthesize both IL-6 mRNAs and protein. Further, we are able to detect IL-6 in the media that decreases over time, with the decrease likely due to its short half-life [17]. Although IL-6 has been detected in the media of primary hepatocytes in culture before [26], it was generally interpreted as likely to be originating from contaminating macrophages. While secretion of IL-6 by contaminating macrophages is certainly a possibility, the paucity of IL-6 in freshly isolated Kuppfer cells versus the abundance observed in hepatocytes would argue against macrophages being the primary source of protein (see Figure 1C). Overall, these findings indicate that hepatocellular IL-6 once produced is secreted and can promote IL-6 signaling, likely in an autocrine manner, although production by hepatocytes may also be important for signaling to other hepatic cell types such as the bile duct epithelium cells [27].

Our data clearly shows that IL-6 can be made by hepatocytes in response to specific stimuli; however, several aspects of the mechanisms regulating hepatocellular-mediated IL-6 production are novel and will merit further study. First, we find that hepatocytes produce IL-6 both *in vitro* and *in vivo* in response to LPS, a factor well known to stimulate production of IL-6 in immune cells via the classic NFκB pathway. While this work demonstrates that LPS-mediated induction of IL-6 can occur in hepatocytes, our data do not support the idea that production in hepatocytes is occurring via a classic NFκB signaling mechanism. Figure 3 clearly shows that only the p65 subunit of NFκB, and not p50, translocates to the nucleus of hepatocytes at one h following LPS injection. This suggests that IL-6 induction by LPS is either occurring downstream of an alternative NFκB pathway or occurs via a completely separate mechanism [28], [29]. Notably, although our data is seemingly at odds with two other studies where NFκB was elevated in liver at 1 h post-LPS injection in whole liver [15] but not in liver with hepatocellular inactivation of NFκB [6], an important consideration is that the other two investigations were performed using gel shift analyses without subsequent supershift confirmation to ensure that both subunits of prototypical NFκB were in the DNA-binding complex. Second, our data indicates that hepatocytes can produce IL-6 in response to HGF both *in vitro* and *in vivo*. In this case, the HGF-mediated stimulation of IL-6 appears to occur in a classic, NFκB-dependent manner (Figure 5), indicating more than one signaling pathway (unknown and classic NFκB) can induce hepatocellular production of IL-6. Third and possibly of most importance, hepatocellular IL-6 induction in response to HGF is exactly the opposite of what we recently observed with regard to HGF and IL-6 in cultured macrophages [10]; in cultured macrophages HGF suppresses production of IL-6, again emphasizing that the mechanisms controlling IL-6 production in hepatocytes are unique from that of macrophages, even when signaling is induced with the same ligand and receptor. In this regard, while certainly not conclusive, the data presented in Figures 6 and 7, combined with studies from the literature, suggest that the source of IL-6 may be relevant for hepatic health, especially with regard to signalling via HGF. Specific removal of MET from mouse hepatocytes led to a marked suppression of IL-6 production after injury (Figures 6C and D), yet global suppression of hepatic MET led to an increase that corresponded with staining of IL-6 in a subset of immune cells (Figures 7B and C). The simplest interpretation of this data is that in the globally suppressed animals, the loss of MET signaling in hepatic immune cells allowed for and enhanced IL-6 production in a subset of cells that contributed to animal survival.

As it now appears that both hepatocytes and Kupffer cells can make IL-6, albeit under different circumstances, this study opens up a greater question regarding whether the cell source of IL-6 is critical within any given context. For example, in the setting of PHx, both HGF [12] and gut bacterial endotoxins (mimicking LPS) participate in the regenerative process [25]. Our current data anticipates a model in which normal functioning livers subjected to hepatic resection are exposed to gut endotoxins that are then able to promote IL-6 production in both hepatocytes and macrophages. Simultaneously, resection-induced induction of active HGF [12] promotes IL-6 production, but in hepatocytes only. As we have previously shown that HGF can suppress IL-6 production in LPS-stimulated macrophages [10], the combined data suggests that context (i.e. induction of endotoxin signaling in the presence or absence of HGF) may regulate which cells produce the IL-6, as well as the quantity being made. Although IL-6 is generally considered a protective cytokine since binding to its receptor has been shown to protect hepatocytes during acute hepatic injury [11], long-term exposure of the liver to IL-6 has paradoxically been demonstrated to be injurious [30]. Hence, an interesting hypothesis would be that during chronic injury, loss of the HGF-mediated suppression of immune cell IL-6 over time results in an enhanced production of immune cell IL-6 that is in part, responsible for the further injuring the liver. Importantly, in this latter scenario, if the loss of MET signaling were due to HGF depletion rather than down-regulation of the receptor, administration of HGF might prove beneficial. In support of this hypothesis, when Kaido *et al*. continuously introduced rat HGF to animals through genetically modified fibroblasts, there was a significantly decreased liver injury following LPS injection [31]. Finally, it is worth noting that the inhibitory effects of HGF on cytokine production in epithelia may be a general mechanism; HGF has recently been shown to inhibit RANTES production in kidney epithelial cells by disrupting NFκB signaling [32]. Hence, in addition to its many other established functions, HGF may also have an innate ability to serve as a general anti-inflammatory molecule for epithelia.

## Methods

### Materials

Experiments involving rats were performed using isolated cells or livers from male Fisher 344 rats. Experiments involving mice were performed using C57Bl/6 or MET Δ mice with a targeted hepatocyte deletion. Unless specified, reagents were purchased from Sigma (St. Louis, MO).

### Animal studies

Ethics statement: All animal procedures were in accordance with the NIH Guide for the Care and Use of Laboratory Animals and were approved by the IACUC at the University of Pittsburgh (protocol number 1009148). For primary cell isolations, hepatocytes and Kupffer cells were simultaneously isolated by an adaptation of the standard 2-step collagenase perfusion [33] as described [34]. Isolated hepatocytes were plated serum-free and allowed to attach on 20% rat-tail collagen-coated plates or cover slips (12 mm) for 2 h prior to fixation or media change with HGF or LPS as indicated. Supernatants containing non-parenchymal cells were pelleted at 200×*g*, resuspended and layered onto a 25–50% Percoll gradient prepared in HBSS (BioWhittaker, Walkersville, MD). Non-parenchymal cells enriched for macrophages were collected and then plated in serum-free DMEM with glucose and L-glutamine (4.5 g/L each; BioWhittaker) for no more than 15 min to prevent adherence of other non-parenchymal contaminants before washing and then either fixing or harvesting for RNA [35]. Purity was verified using an antibody against CD11b (BD Pharmingen, San Diego, CA). All experiments involving primary cells from rat livers were performed a minimum of 3 times; each time point in each experiment shown was plated in either duplicate or triplicate. Hepatectomies in both rats and mice and shRNA knockdown experiments in rats were performed as previously described [24]. For LPS injections, animals were inoculated i.p. with 100 µg LPS/g body weight or a saline vehicle control and livers were then isolated 1 or 4 h post-injection, as described by Maeda *et al* [6].

### Protein isolation, western blots, and slot blot analyses

Lysates were prepared and fractionated as previously described [36]. For slot blot analyses, media from serum-free hepatocyte cultures was removed, flash frozen, and stored until testing. The supernatent was removed at 2 h after plating (attachment) or after an additional 2 h. 5 µl from each well was freshly thawed on ice and assayed per slot, with the subsequent membrane probed as if it were a western blot. Western blots were performed using rabbit anti-IL-6 at a dilution of 1∶1000 (Abcam, ab6672, Cambridge, MA; or Santa Cruz Biotechnology, Santa Cruz, CA), rabbit anti-p50 at 1∶500, rabbit anti-NFκB p65 at 1∶250 (Santa Cruz Biotechnology), and mouse anti-β-actin at 1∶5000. Secondary Abs (Jackson ImmunoLabs, West Grove, PA) were conjugated with horseradish peroxidase. Proteins were visualized on Classic Blue Sensitive X-ray film (Laboratory Products Sales, Rochester, NY) using chemiluminescence reagent (Perkin Elmer LAS, Inc, Boston, MA) according to the manufacturer's directions. To adjust for protein loading differences, bands of interest were normalized to either β-actin or Ponceau protein stain (low-high molecular weight range included) performed on the same blot [37]. ImageJ software (1.38x; NIH, Bethesda, Maryland) was used for image processing and densitometry measurements, and GraphPad Prism version 4.0 (San Diego, CA) was utilized for graph and data analysis (one way ANOVA for group testing with Newman Keuls post-test analyses, one way t-test for paired testing). Results were considered statistically significant if the *P* value was <0.05.

### Immunofluorescent staining

Staining was essentially as described [36]. Cells on cover slips were fixed in 2% PFA at 4°C for 15–30 min, permeabilized with 0.1% Triton-100 (Fisher, Pittsburgh, PA) for 15 min, and blocked with 10% normal donkey serum (Jackson ImmunoLabs, West Grove, PA) in 2.0% BSA for 45 min-1 h before applying primary Ab at dilutions ranging from 1∶50-1∶500 for 1 hr at RT or overnight at 4°C using rabbit anti-IL-6 (Abcam 6672 or 7737, Cambridge, MA), goat anti-NFκB p50 (Santa Cruz; C-19), rabbit anti-NFκB p65 (Santa Cruz; C-20), goat anti-IκB (Santa Cruz), goat anti-lysozyme C (Santa Cruz; C-19), or sheep anti-rat albumin (Bethyl Laboratories, Inc., Montgomery, TX). After washing with 0.5% BSA, cells were incubated with secondary Abs ligated to either Alexa 488, Cy3, or Cy5 (Jackson ImmunoLabs, West Grove, PA), washed, stained with Hoechst to visualize nuclei for 30 s and mounted with gelvatol. Images were taken using epi-fluorescence connected to a digital CCD camera (Olympus Provis, Malvern, NY, USA) or by single slice confocal (Olympus Fluoview 1000). Actin was visualized using Alexa-647-conjugated phalloidin. In each experiment, all exposures were gated the same for comparative purposes. Photoshop CS3 (Adobe Systems, San Jose, CA) or Metamorph Offline version 7.5.4.0 (MDS Analytical Technologies, Sunnyvale, CA) were used for image analysis.

### Immunohistochemistry

Staining was essentially as described [38]. Normal or remnant livers obtained after PHx from rats or mice were fixed for 2 days in 10% formalin and then embedded in paraffin. Immunoperoxidase staining was performed on 4 µm sections using an indirect immunolabeling procedure with an Avidin-Biotin Complex. Rabbit anti-NFκB p50, anti-NFκB p65 (Santa Cruz Biologicals, Santa Cruz, CA) or rabbit anti-IL-6 (Abcam, ab7737, Cambridge, MA) was used as the primary antibody. Briefly, the tissue sections were rehydrated and pretreated with a 3% solution of hydrogen peroxide in deionized water for 10 min at room temperature. The slides then were washed in deionized water and microwaved in citrate buffer (pH 6.0) for 10 min. Slides were blocked with Ultra V block (Thermo Fisher, Pittsburgh, PA) for 5 min, then primary antibody was added and they were incubated for 1 h at room temperature. After washing in PBS with 0.25% Tween, the secondary Ab was incubated at room temperature for 30 min. The slides were washed again in PBS with 0.25% Tween and incubated with Vectastain-Elite ABC (Vector Laboratories, Burlingame, CA) for 30 min at room temperature. The slides were developed with liquid 3,3'-diaminobenzidine (Vector Laboratories) and counterstained with Harris hematoxylin (Anatech, Battle Creek, MI). Images were taken using a Nikon Eclipse E600 microscope connected to a Nikon digital camera (DXM1200) with Nikon ACT-1 software, version 2.63 (Melville, NY, USA). All exposures were gated the same for comparative purposes.

### RNA isolation, cDNA synthesis and PCR

RNA was extracted using RNA-Bee (Tel-Test, Friendswood, TX) according to the manufacturer. After DNase treatment (Worthington, Lakewood, NJ), cDNA for use in RT-PCR (SuperScript III, Invitrogen, Carlsbad, CA) was synthesized from 5 µg RNA by random hexamer priming (Invitrogen). To amplify mature IL-6 mRNA, intron-spanning primers (40 pmole each: F:5-TCAACT CCATCTGCCCTTCAG and R:5-AAGGCAGTGGCTAACAAC, GenBank accession number NM_012589) were utilized in a 50 µl reaction containing half the cDNA plus AmpliTaq Gold polymerase (Applied Biosystems, Foster City, CA). Intron-spanning GAPDH primers served as the positive control (F:5-AGATGGTGAAGGTCGGTGTGAACGG; R:5-AGCCTTGACTGTGCCGTTGAACTTG, GenBank accession number NM_017008.2). An IL-6 intron with comparable G/C content provided a negative control and was generated by performing PCR on sheared genomic rat DNA (primers: 5 pmole each, F:5-GTAAGTGAAGGCAGTTTCTCGCCCT; R:5-CTGCGTGGAGGAAAGGGAAAGAAGC). The IL-6 cDNA was 104-bp; GAPDH was 184-bp and the IL-6 intron was 163-bp. PCR products were run on 2% agarose gels and visualized with AlphaImager 4.1 software (Alpha Innotech, San Leandro, CA) using ultraviolet light. For RT-PCR analyses, ImageJ software (1.38x; NIH, Bethesda, Maryland) was used for image processing, and GraphPad Prism version 4.0 (San Diego, CA) was utilized for graph and data analysis (one way ANOVA for group testing with Newman Keuls post-test analyses, one way t-test for paired testing). Results were considered statistically significant if the *P* value was <0.05.

### Subcloning and labelling for FISH

PCR products were eluted and gel purified using illustra GFX (GE Healthcare, Piscataway, NJ). DNA was ligated with TOPO 2.1 and used to transform Mach1 –T1^R^ *E. coli* (Invitrogen). Transformed colonies were grown in Luria-Bertani broth (1% Tryptone, 0.5% Yeast Extract, 1% NaCl) containing ampicillin and purified plasmid was isolated from the clones using QIAprep spin or Plasmid maxi kit (Qiagen, Valencia, CA). *Eco*R1 (New England Biolabs, Ipswich, MA) digestion was used to verify presence of insert with identity confirmed via sequencing. Inserts were then purified and 1 µg was labelled using ULYSIS nucleic acid labeling kit with Alexa Fluor 546 dye (Molecular Probes, Eugene, OR). After DNAs were purified of excess dye using G-25 columns (GE Healthcare, Piscataway, NJ), labelling efficiency was calculated according to the manufacture's protocol; all probes fell within desired ratios for labelling capacity.

### FISH for mRNA

FISH for the mRNAs (GAPDH and IL-6) were performed using intron-spanning probes to assure detection of mature message. A G/C content matched sequence from the spanned IL-6 intron served as the negative control (see above). All solutions except the balanced salt solution were prepared in 0.1% diethylpyrocarbonate-treated H_2_O. Cultured hepatocytes and Kupffer cells were fixed and hybridized as described previously [39] with some modifications. Cells were fixed in 4% PFA with PBS at 4°C for 10 min, washed with a sterile balanced salt solution (67.0 mM KCl, 1.4 M NaCl, 100.7 mm HEPES, 47.5 mM NaOH) and stored in 70% ethanol at 4°C. At hybridization, cover slips were removed from ethanol, rinsed with 0.5× SSC (1x = 150 mM NaCl, 15.0 mM sodium citrate), and incubated in a moist chamber at 45°C for 1–3 h with hybridization buffer consisting of: 50% formamide, 2.5× Denhardt's solution (0.5% Ficoll 400, 0.5% polyvinylpyrrolidone, 0.5% BSA), 3× SSC, 0.5% SDS, 5% dextran sulphate and 10 mg/ml sheared salmon sperm DNA. Following pre-hybridization, the cells were washed with 0.5× SSC. Denatured, fluorescently labelled cDNA (150 ng) along with hybridization buffer was added to each cover slip and, a larger cover slip was placed over the cells and sealed using rubber cement. Cells were hybridized overnight in a moist chamber at 45°C. Cover slips were then successively washed 3 times for 25 min in 2× SSC, 0.1× SSC and 0.5× SSC (all preheated to 45°C), incubated with Hoechst for 30 s and washed in 0.5× SSC. Slips were then mounted onto slides with gelvatol, examined using epi-fluorescence microscopy and color merged using MagnaFire version 2.1 software (Optronics, Goleta, CA). In each experiment, all exposures were gated the same for comparative purposes.

For FISH to whole tissues, liver was harvested, frozen in OCT (TissueTek, Torrance, CA) and sectioned (5 µm). Cryosections were fixed and pre-treated essentially as previously described [39]. Sections were fixed in 4% PFA with PBS for 20–25 min, incubated twice for 15 min each in PBS with 0.1% active diethylpyrocarbonate, then equilibrated for 15 min in 3× SSC. Sections were exposed to 1µg/ml proteinase K (Novagen, Gibbstown, NJ) for 10 min at room temperature, then hybridized and visualized as described above. In each experiment, all exposures were gated the same for comparative purposes.

In double-label experiments, designed to simultaneously visualize protein and mRNA, immunofluorescent labeling preceded the *in situ* hybridization. Complete controls were always performed for each FISH, immunofluorescent, and double label experiment (some not shown for brevity).
