# Correction: Synthesis of IL-6 by Hepatocytes Is a Normal Response to Common Hepatic Stimuli

**DOI:** 10.1371/journal.pone.0224498

**Published:** 2019-10-23

**Authors:** Callie A. Norris, Mu He, Liang-I Kang, Michael Qi Ding, Josiah E. Radder, Meagan M. Haynes, Yu Yang, Shirish Paranjpe, William C. Bowen, Anne Orr, George K. Michalopoulos, Donna B. Stolz, Wendy M. Mars

The images in Figs 3A and 4C are inadvertently duplicated. Figure 4C includes the correct data, but the 20 ng/ml HGF and No treatment data from Figure 4C are errantly reported as +LPS and -LPS results, respectively, in Fig 3A. The original image files for results reported in Figs 3A and 4C are no longer available, but the authors provide here an updated version of Fig 3 in which panel A includes the correct images from the original experiment. A member of *PLOS ONE*’s Editorial Board reviewed the updated figure and confirmed that it supports the results reported in the original article. Please see the correct Fig 3 here.

**Fig 3:**
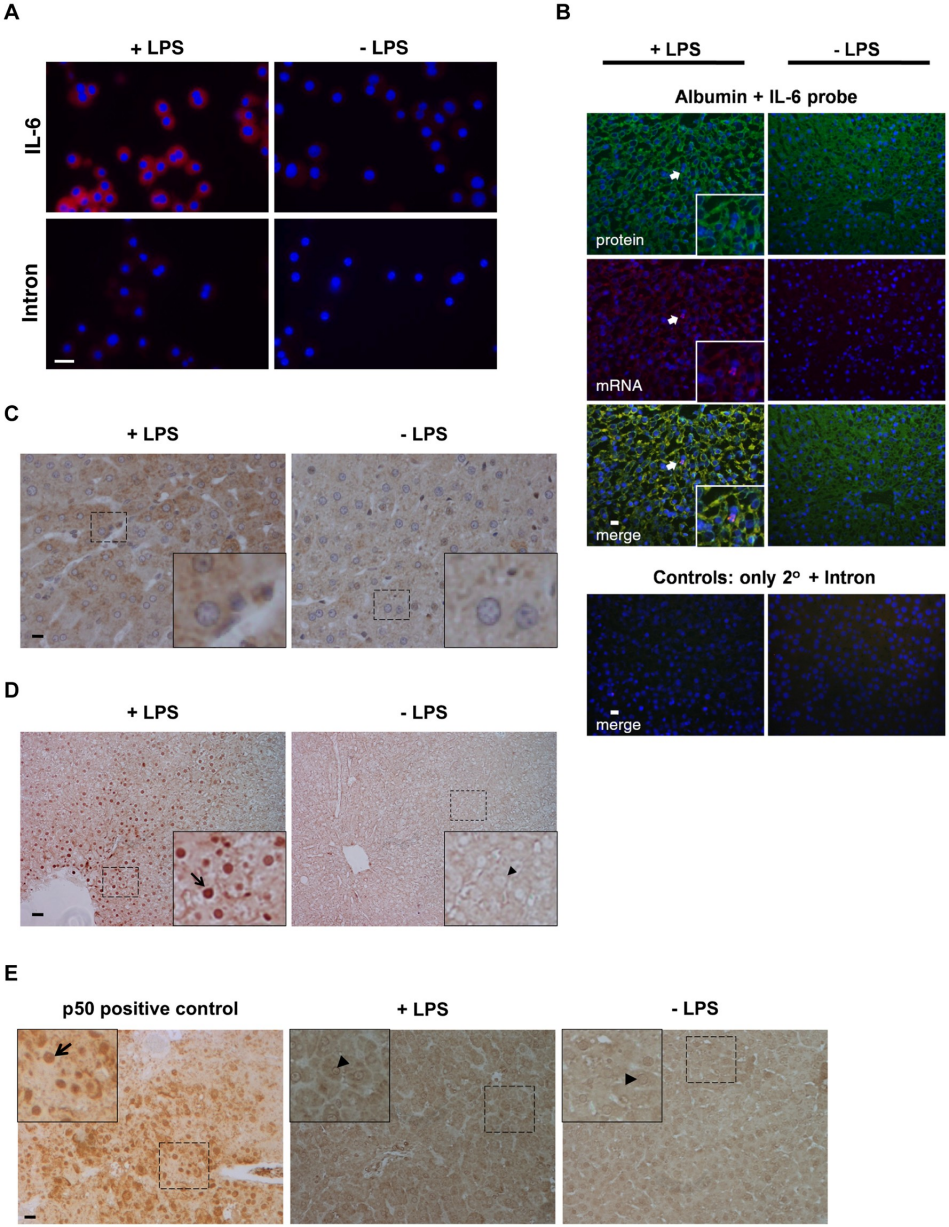
IL-6 synthesis and NFκB signaling in hepatocytes after LPS injection. (A) FISH for IL-6 mRNAs in serum-free rat hepatocyte cultures, 15 min after media change with 1 μg LPS/ml or diluent (control). For these photographs, as a baseline level of IL-6 mRNA was known to be present (see 1D), gating was adjusted with the diluent-treated sample serving as the baseline. (**B, C**) Rat livers were injected with 100 μg/kg LPS or saline (control) and harvested at 4 h post treatment. In **B**, samples were simultaneously stained for albumin protein (green) and IL-6 mRNA (red). Co-localization (merge) appears as yellow. A presumptive inflammatory cell (expressing IL-6 mRNA but albumin-negative) is indicated by an arrow and featured in magnified inserts. Mock hybridizations (not shown) were used as the immunofluorescent gating control. **C** shows standard immunohistochemical staining using an antibody against IL-6. (**D, E**) Representative immunohistochemistry depicting nuclear p65 (**D**), or p50 (**E**) with and without LPS treatment. Arrows show nuclei stained with p65 or p50 (brown), arrowheads indicate unstained nuclei (colorless). Simultaneously stained tissue section from liver of ILK-null mice [40] shows positive nuclear localization staining for p50 (left panel of **D**). Dotted boxes are featured in magnified inserts. Scale bars, 20 μm in all images except for **D** (40 μm).

The raw data used to generate the figures in the article are still available for all figures and will be provided upon request, with the exception of the data used to generate the panels with fluorescent imaging (Figs 1C, 1D, 1E, 2A, 2B, 2D, 2E, 2F, 3A, 3B, 4C, 4E, 5A and 5C).

The authors apologize for the errors in the published article.
